# Supplementary material for: Thalamocortical dysrhythmia detected by machine learning
Source: Nat Commun. 2018 Mar 16;9:1103. doi: 10.1038/s41467-018-02820-0 (PMC5856824; doi:10.1038/s41467-018-02820-0)
Supplement: Supplementary file 1 — Supplementary Information(PDF 809 kb) [file 41467_2018_2820_MOESM1_ESM.pdf]

Supplementary Methods

SVM Classify method

The SVM Classify method used is a multinomial logistic regression model with a ridge estimator to guard against overfitting by penalizing large coefficients [1]. The idea that, if there are k classes for n instances with m attributes, the parameter matrix B to be calculated will be an *m*(k-1)* matrix.

The probability for class j with the exception of the last class is

$$Pj(Xi) = exp(XiBj)/((sum[j=1..(k-1)]exp(Xi*Bj))+1)$$

The last class has probability

$$1-(sum[j=1..(k-1)]Pj(Xi)) = 1/((sum[j=1..(k-1)]exp(Xi*Bj))+1)$$

The (negative) multinomial log-likelihood is thus:

$$L = -sum[i=1..n]\{sum[j=1..(k-1)](Yij * ln(Pj(Xi)))+(1 - (sum[j=1..(k-1)]Yij))* ln(1 - sum[j=1..(k-1)]Pj(Xi))\} + ridge * (B\text{^}2)$$

In order to find the matrix *B* for which L is minimized, a Quasi-Newton Method is used to search for the optimized values of the *m*(k-1)* variables. Note that before we use the optimization procedure, we 'squeeze' the matrix *B* into a *m*(k-1)* vector.

We used multinomial logistic regression model with ridge parameter of 1.0E-8.

Weighting of Data

In order to determine whether imbalanced class sizes play a role in the accuracy of the model retrieved for tinnitus/control, pain/control, Parkinson’s/control, and depression/control data, we

randomly select subgroups of the healthy control subjects (N = 264) similar in size to, respectively, the tinnitus group (N = 153), chronic pain group (N = 78), PD group (N = 31), and the major depression group (N = 15). This weighted dataset was then used to generate a prediction model and to model accuracy values. This was done 100 times and the resulting weighted model accuracy statistics were averaged across all trials and compared to our test (i.e. unbalanced/unweighted) model.

Tinnitus. A comparison between the unweighted model and the weighted model did show a significant different for accuracy rate (F = 33.64, p < .001). However, no significant effect was obtained for the TPR, FPR and ROC between the unweighted and weighted models. A significant effect was obtained for κ-statistic (F = 64.77, p < .001), RMSE (F = 17.47, p < .001), and MAE (F = 13.81, p = .001)(see Fig. 3S).

Pain. A comparison between the unweighted model and the weighted model did not show a significant different for accuracy rate. A significant effect was obtained for the TPR (F = 6.78, p = .013), the FPR (F = 208.67, p < .001) and the ROC (F =10.74, p = .002) between the unweighted and weighted models. A significant effect was also obtained for κ-statistic (F = 69.69, p < .001) but not for MAE and RMSE (see Fig. 3S).

Parkinson. A comparison between the unweighted model and the weighted model did not show a significant different for accuracy rate and the TPR. A significant effect was demonstrated for the FPR (F = 55.53, p < .001), the ROC (F = 8.10, p = .007), an RMSE (F = 92.79, p = .007), between the unweighted and weighted models. No significant effect was also obtained for κ-statistic and MAE (see Fig. 3S).

Depression. A comparison between the unweighted model and the weighted model revealed a significant different for accuracy rate (F = 52.38, p < .001), the TPR (F = 44.59, p <

.001), the FPR (F = 6.43, p = .015), the ROC (F = 119.61, p < .001), the κ-statistic (F = 6.69, p = .014), the RMSE (F = 157.17, p < .001) and MAE (F = 19.66, p < .001) between the unweighted and weighted models (see Fig. 3S).

Overall, the differences obtained between the weighted and unweighted models are minor. For tinnitus and depression we only see an increase in correctly classified subjects of 2.54% and 5.60%, respectively, while no significant effect was obtained for pain or Parkinson's disease. For tinnitus, pain, and Parkinson's, the results are not consistent over the different outcome measures (correctly classified, incorrectly classified, TPR, FPR, ROC, κ-statistic, RMSE, and MAE) of the SVM-learning approach. However, for depression, the weighted model shows a weak yet a significant improvement over all outcome measures.

Supplementary table 1. Anthropometric measures for the non-TCD group (i.e. obese)

	**Obese**	
	M	*Sd*
Body weight (kg)	103.35	17.25
Height (cm)	163.81	4.58
BMI	37.52	5.51
Systolic BP	132.18	11.24
Diastolic BP	81.45	7.02
Heart rate	73.55	8.21
Waist	111.36	12.34

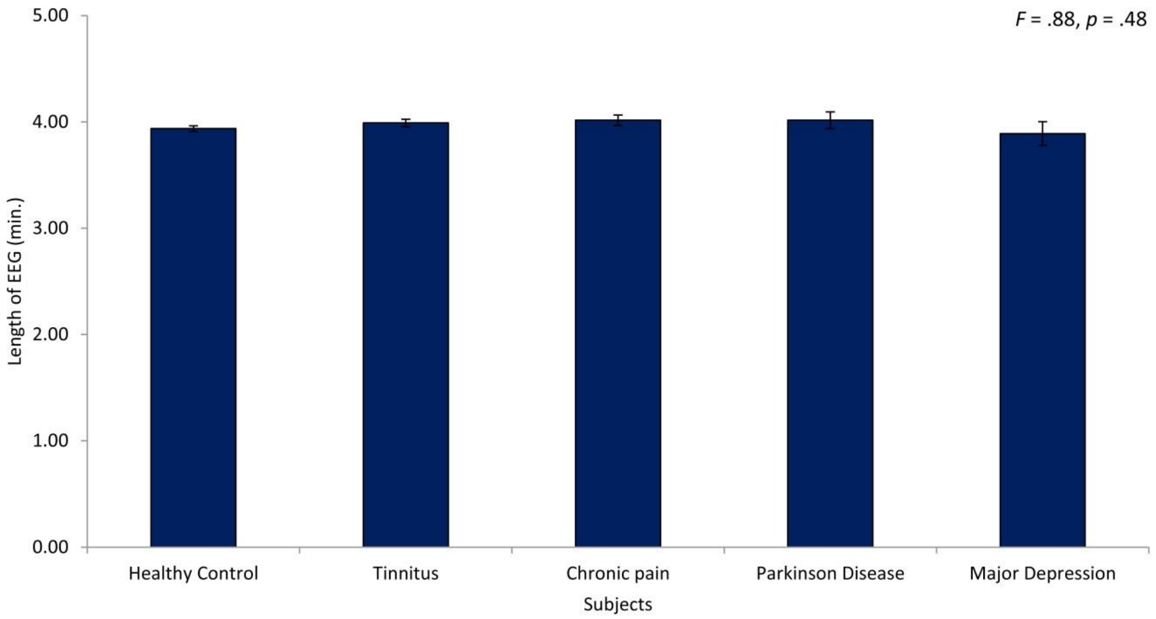

Supplementary figure 1. After artifact rejection, a comparison was made between the different groups (healthy control subjects, tinnitus subjects, subjects with chronic pain, subjects with PD, and subjects with major depression) for the average length of the EEG. This analysis showed no significant difference between the different groups ($F = .88$, $p = .48$)

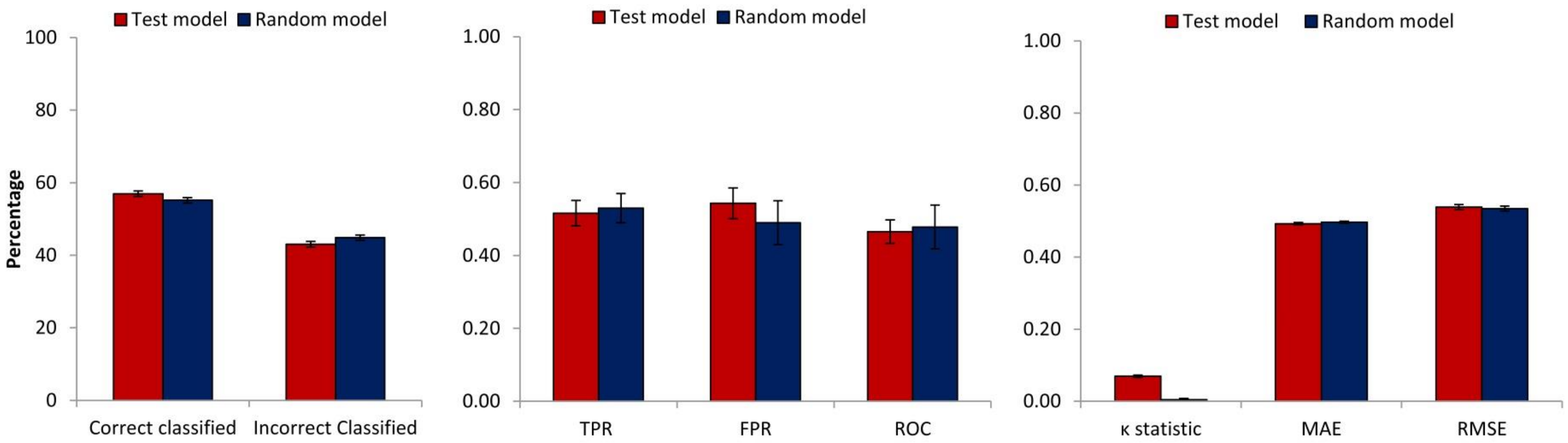

Supplementary Figure 2. Obtained model using support vector machine learning to differentiate between non-TCD patients vs. controls. SVM learning cannot differentiate between the disorder and healthy control subjects in comparison to a random model. The sensitivity of the models, the area under the curve, and the false discovery rate were not significantly different for the obtained model in comparison to the random model. No significant effect was identified by comparing the κ-statistic MAE and RMSE.

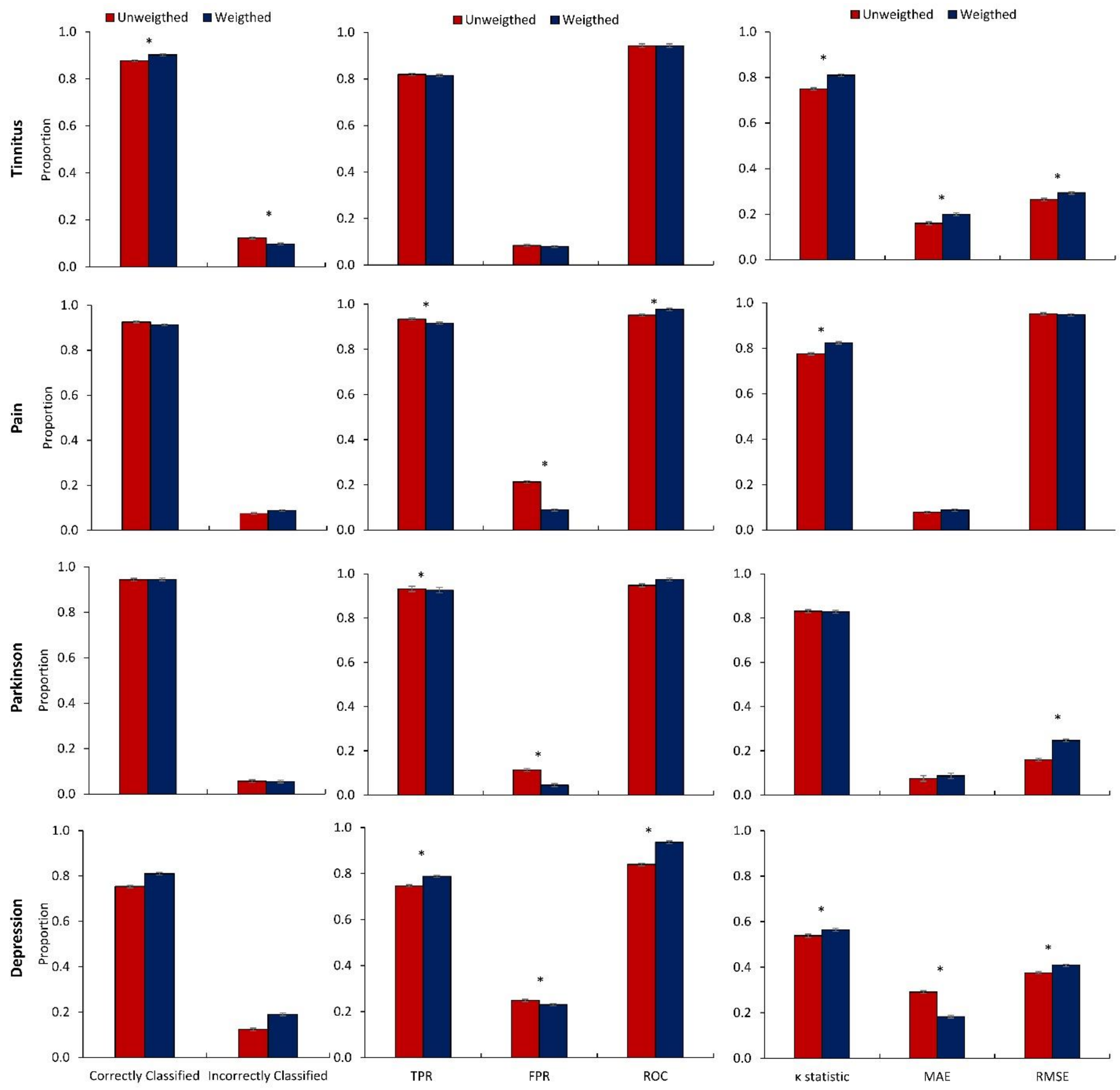

Supplementary Figure 3. Comparison between Weighted and Unweighted models using support vector machine learning to differentiate between respectively tinnitus vs controls, pain vs controls, Parkinson disease vs controls, and depression vs controls. (* indicates a significant effect)

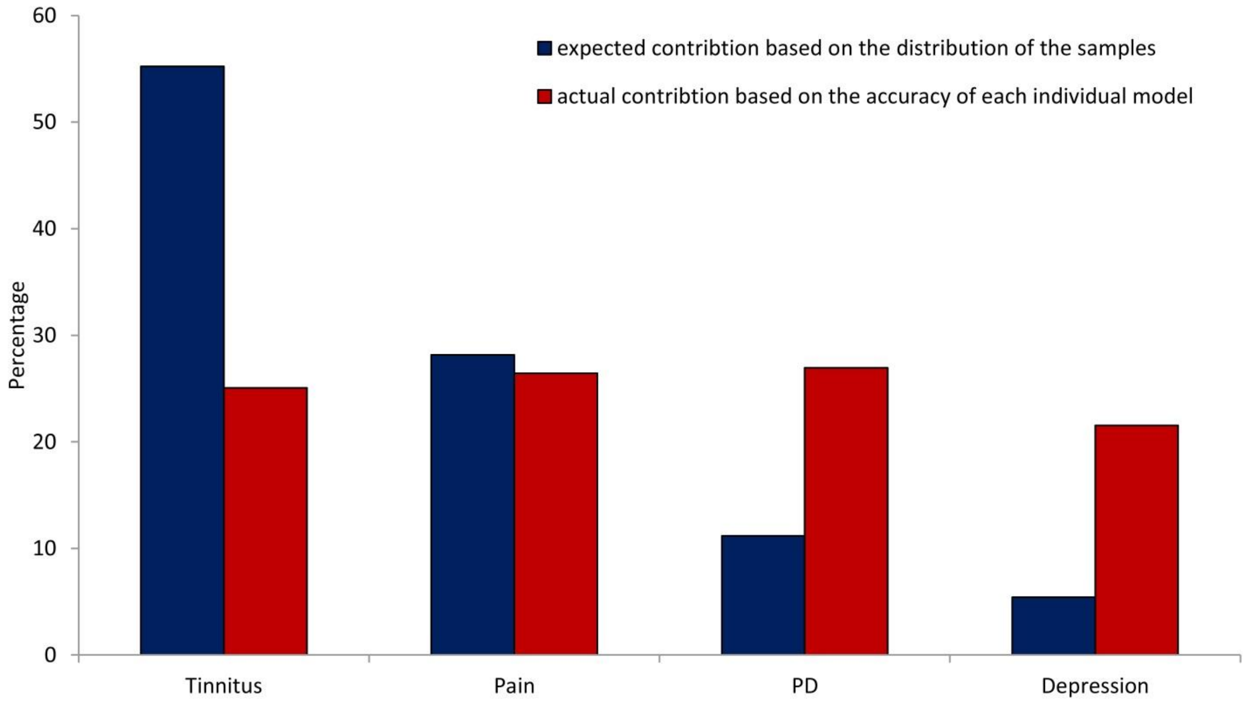

Supplementary figure 4. A comparison between the expected contributions to the full model based on the distribution of the sample of each disorder in comparison to the actual contribution to the full model based on the accuracy of each individual pathology shows that the individual pathologies equally contribute to the full model.

Supplementary References

1 LeCessie, S. & van Houwelingen, J. C. Ridge Estimator in Logistic Regression. *Applied Statistics* **41**, 191-201 (1992).
